# Methyl Eugenol: Its Occurrence, Distribution, and Role in Nature, Especially in Relation to Insect Behavior and Pollination

**DOI:** 10.1673/031.012.5601

**Published:** 2012-04-24

**Authors:** Keng Hong Tan, Ritsuo Nishida

**Affiliations:** ^1^ Tan Hak Heng, 20, Jalan Tan Jit Seng, 11200 Penang, Malaysia; ^2^ Laboratory of Chemical Ecology, Graduate School of Agriculture, Kyoto University, Kyoto, 606–8502, Japan

**Keywords:** allomone, attractant, *Bactrocera*, chemical ecology, floral fragrance, insect pollinators, plant-insect interactions, plant semiochemicals, sex pheromone, synomone, tephritid fruit flies

## Abstract

This review discusses the occurrence and distribution (within a plant) of methyl eugenol in different plant species (> 450) from 80 families spanning many plant orders, as well as various roles this chemical plays in nature, especially in the interactions between tephritid fruit flies and plants.

## 1. Introduction

Plants produce a huge array of chemicals, numbering tens of thousands, primarily for defense against herbivores and pathogens as well as for production of floral fragrance to attract pollinators. Among them is a class of phenolics that consists of a group of compounds known as phenylpropanoids. The phenylpropanoids have numerous functions in plants, ranging from structural constituent, growth, and reproductive biochemistry and physiology to chemoecological interactions with microbes, animals (particularly insects), and neighboring plants.

**Figure 1.**
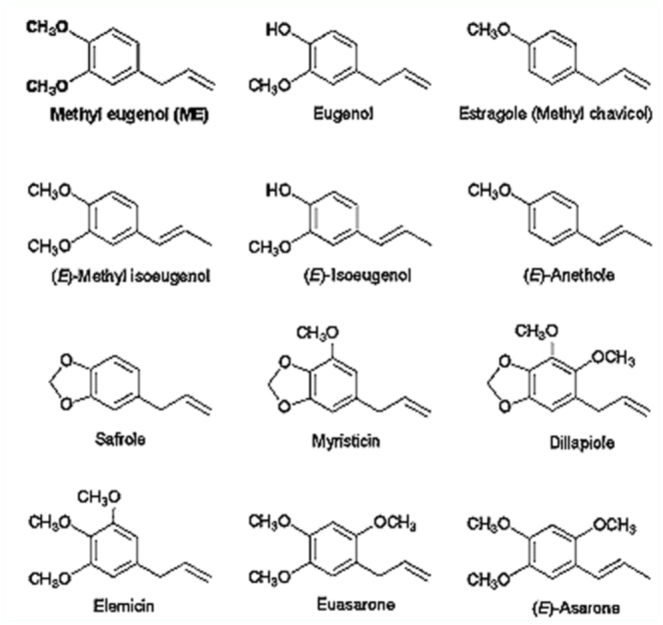
Chemical structures of methyl eugenol (ME) and its analogs. High quality figures are available online.

Methyl eugenol (ME) CAS No. 93-15-12 (Figure 1) is a phenylpropanoid chemical with many synonyms: 4-allylveratrole; 4-allyl-l,2-dimethoxybenzene; eugenyl methyl ether; 1,2-dimethoxy-4-(2-propenyl)benzene; 3,4-dimethoxy-allylbenzene; 3-(3,4-dimethoxyphenyl)prop-l-ene; O-methyleugenol; and methyl eugenol ether. It is directly derived from eugenol, a product from phenylalanine (an essential amino acid) through caffeic acid and ferulic acid via ‘the shikimate pathway’ (Herrmann and Weaver 1999). It is a common phenylpropanoid found in many plant species, particularly in spices and medicinal plants. Furthermore, this chemical can be converted to other useful phenylpropanoids either to elemicin or myristicin, and then, in the latter compound, to dillapiole, via the regulation of two genes in *Perilla frutescens* (Lamiaceae) (Koezuka et al. 1986).

Synthetic ME has been used extensively: a) as a flavoring agent in many types of processed food, soft drinks, and sauces; b) in perfumery; and c) as an essential oil in aromatherapy. From an entomological perspective, synthetic ME has been successfully used in: a) fruit fly surveys (Tan and Lee 1982) and quarantine detection (see reviews by Metcalf and Metcalf 1992; Vargas et al. 2010); b) estimation of native fruit fly populations (Steiner 1969; Newell and Haramoto 1968) and survival rates in natural ecosystems (Tan 1985; Tan and Jaal 1986); c) determining the relationship between fruit phenology and native fruit fly population dynamics (Tan and Serit 1994); d) monitoring movement of native fruit flies between different ecosystems (Tan and Serit 1988); and e) control of tephritid fruit flies (Diptera: Tephritidae) via male annihilation technique through mass trapping (see review by Vargas et al. 2010).

## 2. Methyl eugenol in nature

The role of ME in citronella grass, *Cymbopogon nardus* (Poaceae), in the strong attraction of *Dacus* (currently *Bactrocera*) fruit flies which also visited other plant species including flowers of papaya and *Colocasia antiquorum*, was first discovered almost a century ago (Howlett 1915). Sixty years later, ME was found to be the most active attractant for the oriental fruit fly, *Bactrocera dor salis*, when compared with 34 chemical analogs (Metcalf et al. 1975). Since then, about 20 plant species from 16 families were reported to contain ME, and the role of chemicals as plant kairomone in dacine fruit fly ecology has been discussed (Metcalf 1990; Metcalf and Metcalf 1992). Additionally, eight plant species containing 0.1–17.9% ME as a natural constituent, and another seven plant species with ME but without quantitative data, were reported by De Vincenzi et al. (2000). Prior to this review, it was reported that a) ME was present in 20 angiosperm and 3 gymnosperm families (Schiestl 2010); and b) ∼350 plant species belonging to 61 families possessed ME as a constituent component and/or as a component of floral fragrance (Tan et al. 2011).

### 2.1. Occurrence of methyl eugenol

From an intensive literature search conducted over the first half of 2011, an additional ∼100 were added to the 350 plant species to yield a total of over 450 species from 80 families spanning 38 plant orders that contain varying amounts of ME in essential oils from leaves, roots, stems, flowers, or whole plant extracts. The compiled species are presented here in two separate tables. Table 1 shows over 370 species of plants listed alphabetically from 62 families (one fern, two gymnosperms, four monocots, and 55 dicots) having ME content varying from a trace quantity to 99% of essential oils detected in various plant organs, except flowers (which will be presented in Table 2 in section 3.4). The large number of families involved indicates that biosynthesis of ME evolved independently in many of the Plantae orders and families. Families that are represented by 10 or more species in Table 1, in decreasing order, are Asteraceae (47), Apiaceae (44), Lamiaceae (38), Lauraceae (34), Aristolochiaceae (32), Rutaceae (23), Myrtaceae (20), Poaceae (12), Cupressaceae (10), Euphorbiaceae (10), and Zingiberaceae (10). The ME content varies greatly within and between species as well as within and between the plant families. Several species have ME content over 90% in essential oils, namely *Croton malambo* (Euphorbiaceae), *Cinnamomum cordatum* (Lauraceae), *Melaleuca bracteata, M*. *ericifolia*, *M*. *leucadendra*, *M*. *quinquenervia*, *Pimenta racemosa* (all Myrtaceae), *Piper divaricatum* (Piperaceae), and *Clusena anisata* (Rutaceae). Furthermore, 68 species possess ME content between 20 and 90% in essential oils of either a whole plant or a part thereof (Table 1). These plant species are likely to involve ME in their chemical defense against pathogens and/or insect herbivores. Most of the plant species listed in the table are either spices, medicinal plants (many with ethnopharmacological properties), or plants of economic importance, especially in the production of essential oils for aromatherapy and perfumery. As such, many more plant species, currently with little or no anthropocentric importance, may contain ME and await discovery and/or chemical analysis.

Methyl eugenol, as a constituent in leaves, fruits, stems, and/or roots, may be released when that corresponding part of a plant is damaged as a result of feeding by an herbivore. If present in sufficiently high concentration, it will immediately deter the herbivore from further feeding on the affected part (see section 3.2.3). In this case, ME acts as a deterrant or repellant. In many plant species, ME is present along with varying amounts of eugenol—ME's immediate precursor (see section 3.4.2.2 B). Both the compounds are found in most spices.

For plant species with low ME content, this component may be detected only in certain developmental stages. This is demonstrated by the sweet marjoram, *Origanum majorana* (Lamiaceae), in which ME was detected during the early vegetative and budding stages of four growth stages investigated (Seilami et al. 2009). Similarly, ME was detected in *Artemisia abrotanum* (Asteraceae) only during the emergence of runners and mass flowering phases among four studied (Table 1). Nevertheless, in *Artemisia dracunculus* ME was detected at 6.06, 6.40, 38.16, and 7.82 % of essential oil weight during emergence of runners, budding, mass flowering, and seed ripening phases, respectively (Khodakov et al. 2009).

A native Mediterranean plant species with ethnopharmacological properties, *Erodium cicutarium* (Geraniaceae), was shown to contain a relatively high content of ME (10.6%) in leaf hexane extract (Lis-Balchin 1993). Nevertheless, out of approximately 170 chemical components, many of which existed in trace quantities, ME was not detected in some specimens of the same species (Radulovic et al. 2009). This finding probably reflects geographical variation among varieties or populations and not different extraction methods or chemical analyses.

High variation within a plant species in terms of ME content may lead to the identification of distinct chemotypes. To further illustrate varietal differences in plant species, two common *Ocimum* species (Lamiaceae), *O*. *basilicum* and *O*. *sanctum*, which are frequently used for culinary and medicinal purposes in Southeast Asian countries in particular, show distinct variations in terms of ME content. 19 accessions/varieties of *O*. *basilicum* (sweet basil), two wild and 14 cultivated as ornamentals in Sudan, two from Germany, and one from United Arab Emirates, had varying contents of phenylpropanoids—eugenol, ME, and methyl cinnamate—in combined leaf and flower essential oils. As indicated by peak area in essential oils, 12 varieties had highly variable content of eugenol from 0.05 to 43.3%, and for methyl cinnamate, 11 varieties had content from 1.9 to 42.4%, of which seven had over 15%. However, only one variety had 8.7% ME without the other two phenylpropanoids, and three had ME in trace amounts (Abduelrahman et al. 2009). Nevertheless, two varieties of the sweet basil found in Malaysia had no eugenol, but ME content was at 5.6-12.3% in leaf and 3.2-11.1% in inflorescence essential oils (Nurdijati et al. 1996).

*Ocimum sanctum* (holy basil) also varies considerably in terms of ME and eugenol contents in leaf and inflorescence essential oils. Seven varieties of holy basil in Malaysia and Indonesia can be grouped into three chemotypes based on the phenylpropanoid content in leaf essential oils: two as eugenol chemotypes with 66–73% eugenol and 0.5–3.1 % ME, four ME chemotypes with 78–81% ME and 2.7–5.8 % eugenol, and one ME—eugenol chemotype with 52% ME and 27% eugenol (Nurdijati et al. 1996). The phenylpropanoids in the leaves of both sweet and holy basils are not released naturally. They are stored in the numerous oily glands (characteristic of Lamiaceae (formerly Labiatae)). More glands per unit surface area are found on the lower surfaces of leaves in the basils. Healthy leaves on a plant do not attract male *Bactrocera* fruit flies (see 3.3.1. Insect attractant). However, when any part of the plant (especially the leaves) is damaged or squashed, many male fruit flies are attracted to the damaged part, indicating the release of ME and eugenol. Further, it is very interesting to note that the *O*. *sanctum* leaf (chemotype unspecified) essential oil has lipid–lowering and anti—oxidative effects that protect the heart against hypercholesterolemia in rats fed with a high cholesterol diet (Suanarunsawat et al. 2010).

Additionally, another species of *Ocimum* in Brazil, *O*. *selloi*, has two chemotypes. Leaf and flower essential oils of chemotype A contained estragole (methyl chavicol) at 80.7 and 81.8% with ME at 0.79 and 1.13% of peak area, respectively, while chemotype B had ME as the major component at 65.5 and 66.2% of peak area in leaf and flower essential oils, respectively, and with no trace of estragole (Martins et al. 1997).

The same species of plant grown in different countries may show high variation in chemical constituents. This was well illustrated by *Alpinia* speciosa (Zingiberaceae) in which leaves collected from Japan contained ME, estragole, and (E)methyl cinnamate at 2.9, 4.6, and 24.1% of essential oil. The phenylpropanoids were not detected in leaves that originated from Amazonia (Brazil), Martinique (French West Indies), Rio Grande (USA), and China and Egypt (Prudent et al. 1993).

Furthermore, within a variety of a plant species, the quantity of ME may also vary depending on the plant tissue and on the time of harvest. This is elucidated by *Myrtus communis* var. *italica* (Myrtaceae) grown in Tunisia. The quantity of ME varied from 0.4 to 1.9% of leaf essential oil, with > 1% for October, November, and March over a period of 12 months. The monthly ME content of stem oil varied between 0.8 and 3.6%, with January and April > 3%. However, fruits had monthly ME content of 1.1–1.3% for August and September, which then rose to 3% in subsequent months and remained between 3.1–3.6% from October to January (Wannes et al. 2010).

Even during storage, the major components of essential oils may change considerably. This is shown by *Agastache foeniculum* (Lamiaceae), which contained five major components. During storage of the plants for 17 days, estragole decreased from 63.2 to 50%, with a corresponding increase of ME from 28.6 to 41% in plant essential oil (Dimitriev et al. 1981).

**Figure 2.**
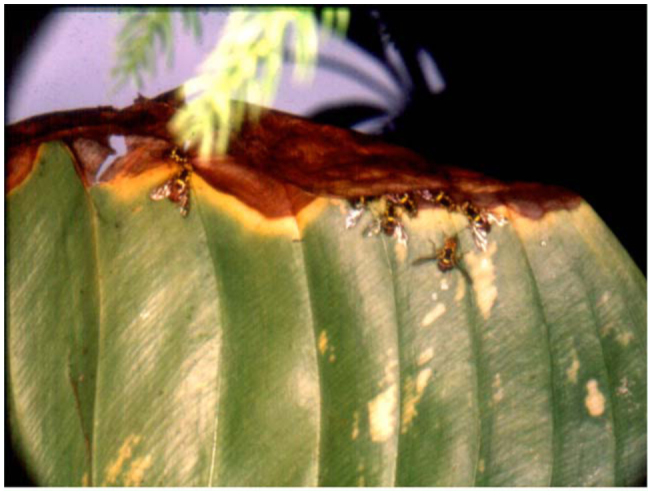
Male fruit flies (*Bactrocera dorsalis* and *Bactrocera umbrosa*) feeding along yellow—brown border of an infected leaf of *Proiphys amboinensis.* High quality figures are available online.

It was shown that green parts of *Proiphys amboinensis* (Amaryllidaceae) leaves contained a trace quantity of ME, and during browning of a leaf, the yellow and brown parts contained 0.1 and 0.2–0.3 µg/mg of leaf, respectively, that attracted many male fruit flies (Chuah et al. 1997). The attraction phenomenon has never been observed in the normal browning of the leaves, except on one occasion after a raining shower when an infected leaf attracted many male fruit flies(ME—sensitive *Bactrocera* species) that fed along a yellow—brown border between the green and yellow to brown parts (Figure 2, unpublished observation). The attractant in the browning phenomenon may be induced or produced by microbes as a result of an infection, and this certainly warrants further investigation.

Besides large variation within species, differences between species within a genus frequently occur. For example, the genus *Heterotropa* (Aristolochiaceae) possesses species with ME content ranging from 0.1 to 50% of volatile oil. Many of the 27 species have ME content below 5% of volatile oil, except for *H*. *fudzinoi* (11%), *H*. *muramatsui* (20%), and *H*. *megacalyx* (50%). Eleven species of *Artemisia* (Asteraceae) have ME in trace quantities (e.g., *A*. *campestris),* whereas *A*. *dranunculus* has an ME content of 35.8%. Similarly, high variation in ME content exists for genera *Ocimum* (Lamiaceae), *Cinnamomum* (Lauraceae), and *Melaleuca* (Myrtaceae), in which most species are known to have relatively high ME content (Table 1). Strangely, many species in the genus *Croton* (Euphorbiaceae) contain ME in aerial parts (stems and leaves) except *Croton microns,* which has ME in flowers but not in leaves (Compagnone et al. 2010). It was found that shading from the direct sunlight also affected the content of phenylpropanoids in leaves. *Ocimum selloi* seedlings from the same population grown under normal sunlight and two different shadings, blue and red, showed a change in two phenylpropanoids, estragole and ME. The leaf estragole content under full sunlight, blue shading (with transmittance of 400–540 nm), and red shading (with transmittance of > 590 nm), was 93.2, 87.6, and 86.1% (relative percentage of peak area), respectively. While for leaves, the ME content was 0.6% under full sunlight and 1.1% under both types of shading (Costa et al. 2010).

### 2.2 Distribution of ME in various plant organs

The distribution of ME among plant organs is never even as illustrated by many of the species listed in Table 1. A Brazilian folk medicine plant, *Kielmeyera rugosa* (Caryophyllaceae), possesses ME only in flowers and not in leaves and fruits; the showy flowers are pollinated by large bees (Andrade et al. 2007). *Valeriana tuberosa* (Valerianaceae), a medicinal plant used as a mild sedative, commonly found in Greece, has eugenol and ME in similar quantities (∼0.45% of oil) in inflorescences but none in roots, stems, or leaves (Fokialakis et al. 2002).

Another medicinal plant, bay laurel *Laurus nobilis* (Lauraceae), is known to have antibacterial, antifungal, anti-inflammatory, and anti—oxidative properties. It was reported to contain ME in all its aerial parts but in different quantities, such as 3.1, 11.8, 4.7, and 16 % of flower, leaf, bark, and wood essential oils, respectively (Fiorini et al. 1997). Recently, 10 populations of wild bay laurel found in Tunisia had ME at 13.1–33.6, 6.6– 17.8, 1.0–16.8, and 3.9–14.3 percentage composition of essential oil in stems, leaves, buds, and flowers, respectively (Marzouki et al. 2009). In another study on the same species, plants from Turkey had ME content that varied considerably between old and young leaves at 1.2 and 0.2% of volatile composition, respectively, while buds had 0.3% and fruits had 0.1% ME, with no ME detected in flowers (Kilic et al. 2004). Additionally, flowers of *Myrtus communis* var. *italica* (Myrtaceae) contained ME at 4.02% of the essential oil as one of seven major components, but as a minor component in leaves and stems at 0.38 and 0.22% of the essential oil, respectively (Wannes et al. 2010)

The amount of ME emitted from flowers of carob tree, *Ceratonia siliqua* (Fabaceae), varies considerably. Whole hermaphrodite flowers did not emit ME, male flowers emitted 2.8% ME of total volatiles, and female flowers of cultivars Galhosa and Mulata emitted 32 and 1.5% of total volatiles, respectively. In this species, the stamens and stigmas did not emit ME, but the nectar disk (source of most volatiles) of hermaphrodite, male, and female flowers emitted 0.8, 1.7, and 4.7–5.7% ME of total volatiles, respectively (Custodio et al. 2006). Whole flowers of *Clarkia breweri* from some plants emit eugenol, isoeugenol, ME, and methyl isoeugenol, while those for other plants do not emit ME and methyl isoeugenol. For flowers that emit all the four phenylpropanoids, the petals emit on average ME, methyl isoeugenol, and eugenol approximately 2.5, 1.8, and 0.5 µg/flower/24 hours, respectively, without any isoeugenol. In contrast, pistils and stamens emit only a single component of methyl isoeugenol and ME in very low quantities (Wang et al. 1997). This and the preceeding examples clearly show that the phenylpropanoids are distributed or released unevenly among different parts of individual flowers. All these species show that distribution or release of ME varies even in different parts of individual flowers.

Fruit of *Myrtus communis* var. *italica* showed variation in many of its 48 volatile components during development and ripening. As to its ME content, it increased slightly during the initial stage of development when the fruit was green in color from 1.14 to 1.26 % (wt/wt) during 30 to 60 days after flowering. Then, ME concentration increased two-fold when the fruits were pale yellow from 3.05–3.30% during 90–120 days after flowering. A slight increase was noted when the fruits ripened and turned dark blue (Wannes et al. 2009).

Calamus or sweet flag, *Acorus calamus* (Acoraceae), is a unique medicinal plant in that, unlike many other species in which ME is mainly found in aerial parts, it has ME in the roots. In this species, aerial parts contained only about 1% ME but root essential oil contained up to 80% ME, particularly in the European and Japanese samples (Duke 1985). In this species, the high ME content may be used as chemical defense against root-feeding insects or nematodes.

The distribution of ME within a plant is clearly uneven. In many species, ME may be detected in a specific plant part but not in other parts. Intraspecific chemical variation may be the result of several phenomena, namely: a) adaptation to different pollinator species, b) random genetic drift, c) adaptation to disruptive learning processes in pollinators among non—rewarding flowers, and d) introgression effects involved in hybridization (Barkman et al. 1997). Another possible phenomenon is the selection pressure exerted by herbivores, microbes, and nematodes in their interactions with plants (see section 3.2).

## 3. Role of methyl eugenol in plants

There are two main theories on the evolution of secondary plant metabolites. First, due to oxidative pressure and the possibility of photo—damage, plants might have developed secondary plant metabolites with antioxidant properties, namely flavonoids, to prevent cellular damage by highly reactive chemicals (Close and McArthur 2002; Treutter 2005). The second theory states that it arose from the relationship between plants and various groups of herbivores or pathogens (Dicke and Hilker 2003; Franceschi et al. 2005), and this latter view is further substantiated in this review.

### 3.1. Induction of phenylpropanoid biosynthesis due to stress

Phenylpropanoids form a large subclass of chemical compounds within the class of phenolics. All of them are derived from cinnamic acid/p—coumaric acid, which in turn is derived from phenylalanine, an essential amino acid, catalyzed by an enzyme, phenylalanine ammonia lyase (see 3.4.2.B below). This enzyme is the branch—point enzyme between primary (shikimate pathway) and secondary (phenylpropanoid) metabolisms. Many simple and complex phenylpropanoids may be induced in plants by external stresses, such as high ultra—violet light, pathogen attack, and physical —wounding, such as that caused by herbivory (see review by Dixon and Palva 1995). The cytochromecytochrome-p450s-dependentp450s-dependent oxygenases, belonging to a large plant gene family, are involved in primary metabolism, such as in steroid and phenylpropanoid biosynthesis, and secondary metabolism. A similar phenomenon also exists for O-methyltransferase enzymes that are involved in primary metabolism, namely lignin synthesis and secondary metabolism, such as phenylpropanoid biosynthesis (Pichersky and Gang 2000).

Essential oils of three untreated orange varieties of *Citrus sinensis* (Rutaceae)— Hamlin, Pineapple and Valencia—did not contain any ME. But, when treated with abscission agents to loosen fruits for mechanical harvesting, six phenylpropanoids, namely eugenol, ME, (E)- and (Z)-methyl isoeugenol, elemicin, and isoelemicin, were detected for the first time. Among these compounds, ME was the most abundant component present at 42 ppb in orange juice from the treated fruits (Moshonas and Shaw 1978). This study clearly shows induction of phenylpropanoid biosynthesis in fruit under stress. The role of ME in the treated orange is unclear, however.

### 3.2. Defense

Plants produce a large diversity of chemical compounds to deter phytophagous organisms, especially against insect herbivores and/or pathogens. These chemicals may exist as plant primary constituents or as secondary byproducts/metabolites. They have diverse biochemical and physiological activities against a) pathogenic microbes, b) competitive/neighboring plant species, and c) herbivores. Plant chemical constituents that are not secreted naturally, and affect animal behavior in self—defense by acting as a toxicant, antifeedant, deterrant, irritant, repellant, and/or growth regulator, act as para—allomones (an allomone is a naturally secreted chemical that benefits only the releaser in an interaction between two species of organisms).

### 3.2.1. Microbes.

Essential oils and ME have been known for a long time to possess antifungal activity. ME and eugenol have similar antifungal activity against seven species of fungus at 2.0 mM concentration (Kurita et al. 1981). The essential oil of *Echinophora sibthorpiana* (Apiaceae) contains ME, and the oil (∼0.1%) or ME alone (at 0.05–0.1%) showed some inhibitory activity against fungi and bacteria (Kivanc 1988). At temperatures 5–15 °C, 1000 ppm ME delayed mold's initiation of mycelium and spore development in 32 strains: four of *Aspergillus ochraceus*, two *A*. *niger*, 16 *Penicillium clavigerum*, and 10 *P*. *expansum* (Kivanc and Akgul 1990). Furthermore, sprays of 0.5% ME on peanut pods and kernels prevented colonization of *Aspergillus flavus*, common mold, and inhibited aflatoxin synthesis in the fungus. Consequently, it was suggested that ME be used to prevent infestation of the fungus in peanuts (Sudhakar et al. 2009).

Fruit essential oil of emblica, *Phyllanthus emblica* (Euphorbiaceae), that contained 1.25% ME among eight major components had high antimicrobial activity against contaminating microbes, such as: a) Gram— positive bacteria, e.g., *Bacillus subtilis* and *Staphylococcus aureus*; b) Gram—negative bacteria, e.g., *Escherichia coli*, and *Salmonella*; c) molds, e.g., *Aspergillus niger* and *A*. *oryzae;* and d) the budding yeast, *Saccharomyces cerevisiae*. The antimicrobial activity of the oil was mainly due to the presence of ME, β-caryophyllene, βbourbonene, and thymol (Zhao et al. 2007). Recently, another fruit essential oil of *Eugenia singampattiana* (Myrtaceae) had major constituents, namely, a-terpineol (59.6%), camphene (12.1%), ME (11.5%), and αpinene (4.7%). A minimum inhibitory concentration (MIC) at 0.2 µL/mL of the essential oil yielded complete inhibition against *Candida albicans* (a form of yeast that causes infections such as “thrush”) (Jeya Johti et al. 2009).

The growth of a strain of *Campylobacter jejuni*, a major bacteria species causing gastroenteritis in humans worldwide, was inhibited by essential oil of carrot, *Daucus carota* (Apiaceae), as well as individual component of ME and elemicin at a MIC of 250 µg/mL, which was slightly less effective than methyl isoeugenol at MIC of 125 µg/mL (Rossi et al. 2007).

### 3.2.2. Nematodes.

The pinewood or pine wilt nematode, *Bursaphelenchus xylophilus*, is very damaging to matsutake mushroom cultivation in addition to causing pine wilt. Nematicidal activities against the nematode were demonstrated with LC50 (lethal concentration that induces mortality in 50% of test organisms) values for geranial, isoeugenol, methyl isoeugenol, eugenol, and ME at concentration of 0.120, 0.200, 0.210, 0.480, and 0.517 mg/mL, respectively (Park et al. 2007).

### 3.2.3. Antifeedant.

Plant ME in the growing bud of *Artemisia* capillaries was found to inhibit feeding (100% antifeeding activity on 2 cm diameter leaf disc) by larvae of the cabbage butterfly, *Pieris rapae* subspecies *crucuvera* (Katsumi 1987). In addition, ME was the most potent of seven eugenol analogs in essential oil of *Laurus nobilis* against a noctuid moth white—speck, *Mythimna unipuncta* (Muckensturm et al. 1982).

A fresh water aquatic plant *Micranthemum umbrosum* (Scrophulariaceae) possesses elemicin, a phenylpropanoid as one of two chemicals used in chemical defenses against herbivores, which acts as an antifeedant against generalist consumers such as crayfish (*Procambarus acutus*). To determine the structure—activity relationship among eight naturally occurring phenylpropanoids, bioassays were conducted and showed that ME was most active and much more effective than either eugenol or elemicin in deterring feeding by crayfish (Lane and Kubanek 2006).

### 3.2.4. Insects.

Of the nine major constituents of essential oils, benzene derivatives (eugenol, isoeugenol, ME, safrole, and isosafrole) are generally more toxic and repellent to the American cockroach, *Periplaneta americana*, than the terpenes (cineole, limonene, pcymene, and a-pinene). Furthermore, ME was most effective in terms of knockdown activity, as well as repelling and killing effects (Ngoh et al. 1998).

Toxicity of ME against larvae of the tobacco armyworm, *Spodoptera litura*, was found to be significant. Larvicidal activity of a residual ME (15 µg/leaf cm^2^) was 36.0 ±15.3% and 76.6 ±11.5% for 24 and 48 hours of exposure, respectively (Bhardwaj et al. 2010). However, as to mosquitocidal impact, ME, found only in leaves of *Magnolia* salicifolia (Magnoliaceae), induced 100% mortality at 60 ppm against 4^th^ instar larvae of the yellow fever mosquito, *Aedes aegypti*, which is responsible for the spread of dengue fever and Chikungnya viruses (Kelm et al. 1997).

In a fumigation study comparing the toxicity of more than a dozen monoterpenes against the rice weevil, *Si tophi lus oryzae* (Coleoptera: Curculionidae), ME and eugenol were moderately toxic compared to the most toxic compound tested, menthone (Lee et al. 2001). The latter was the main chemical component in *Mentha arvensis* (Lamiaceae) var. *piperascens* essential oil, which in turn was the most toxic among 16 medicinal and spice plants tested. Nonetheless, ME was the most potent inhibitor against the acetylcholine esterase (Lee et al. 2001), an enzyme responsible for the hydrolysis of the neurotransmitter acetylcholine, which can eventually lead to paralysis. Similarly, fruit essential oil of *Illicium simonsii* (Aquifoliaceae) that contained βcaryophyllene (10.30%), δ-cadinene (9.52%), and ME (8.94%) as major components had strong fumigant and contact toxicities against adults of the maize weevil, *Sitophilus zeamais*, with LC50 values of 14.95 mg/L air and 112.74 µg/adult, respectively (Chu et al. 2010). Fumigant and repellant effects, leading to almost 100% mortality within 24 hours, were observed on adult brown plant hoppers, *Nilaparvata lugens*, feeding on rice seedlings placed over a filter paper containing ME residue at ∼0.15mg/cm^2^ (Tan, unpublished data).

It is interesting to note that ME as a fumigant was also very toxic to two global pest fruit fly species—the Mediterranean fruit fly, *Ceratitis capitata*, and the melon fly, *Bactrocera Cucurbitae* (a cue—lure/ raspberry ketone [RK] responsive species)—compared with basil oil, linalool, estragole, and (E)-anethole, all of which showed no knockdown effect at 0.75% concentration (Chang et al. 2009). After two hours of exposure to ME at concentrations of 0.5 and 0.75%, mortality/ knockdown was 96 and 100% against *C*. *capitata* and 98 and 97% against *Ba*. *Cucurbitae.* However, ME was less toxic as a fumigant, even though it was a strong attractant, to the oriental fruit fly, *Ba*. *dorsalis.* Concentrations of 10–100 % induced 35–53% mortality/knockdown against this species (Chang et al. 2009).

### 3.3. Chemical cue

Certain insect species have adapted to using ME as a stimulant or attractant to locate plant host or source for pharmacophagy (consumption of non—nutritive and nonessential chemicals).

### 3.3.1. Insect attractant.

Some insect species are known to be attracted to ME for unknown reasons, while others may be attracted and stimulated to undergo pharmacophagous feeding.

#### *3.3.1.1. Pest insect species.*

Two scarabid pest species, *Cetonia aurata aurata* and *Potosia cuprea*, were captured in traps baited with a known attractant consisting of ME, 1-phenylethanol, and (E)-anethole (1:1:1). However, the numbers trapped were significantly increased for both the species with the addition of a synergist, either geraniol or (+)-lavandulol (Vuts et al. 2010). Larvae of the rice stem borer, *Chilo suppressalis*, are attracted to “oryzanone” (pmethylacetophenone), and ME among 30 compounds related to the “oryzanone” also attracted the larvae (Kawano and Saito 1968). Although ME is not present in rice plants, it may be interesting to evaluate the impact of ME on stem borer physiology and behavior.

Two *Dacus* (currently *Bactrocera*) (Diptera: Tephritidae) species of fruit flies were first discovered to be attracted to citronella grass *Cymbopogon nardus* used as a mosquito repellant (Howlett 1912). Subsequently, ME was positively demonstrated to be solely responsible for the attraction (Howlett 1915). Since then, voluminous publications related to fruit fly attraction to ME have appeared. It should be pointed out at this juncture that all *Bactrocera* species may be categorized into three groups based on their response to two potent attractants: cue—lure, a synthetic analog of RK (195 species cue—lure responders, this chemical being a synthetic of RK) and ME (∼84 ME responders), and non—responders to the attractants (28 species confirmed and 258 species listed under “lures unknown”) (IAEA 2003). The effects of the attractants on sexual behavior of *Bactrocera* fruit flies have recently been reviewed (Shelly 2010).

ME acts as a precursor or booster to male fruit fly sex pheromonal component(s) in the rectal gland of certain *Bactrocera* species (Nishida et al. 1988, 1990, 1993; Tan and Nishida 1995, 1996, 1998). Plant ME, when released, attracts only male fruit flies, although there are two reports of wild females being attracted into traps baited with poisoned synthetic ME (Steiner et al. 1965; Verghese 1998). The attraction of females was probably due to a chemical contamination—perhaps male sex pheromonal components from spontaneous ejaculation induced by the poisoned bait prior to death of captured males. In contrast, no female *Bactrocera dorsalis* or *Ba*. *umbrosa* was ever attracted to or captured in ME— baited clear—traps, without an insecticide, used in the ‘capture—mark—release—recapture’ technique to capture thousands of live wild males for ecological and population studies in areas with high fruit fly infestation (Tan 1985; Tan and Jaal 1986; Tan and Serit 1988, 1994). These field studies further confirm that pure ME is a male attractant, although ME did induce an electrophysiological response in the antennae of *Ba*. *dorsalis* females (Siderhurst and Jang 2006) that may be translated into a negative rather than positive attraction response under natural conditions. Male fruit flies do not directly cause harm or damage to plants by just feeding on ME.

Several putative and ME—sensitive sibling species of the *Bactrocera dorsalis* complex, such as *Ba*. *carambolae*, *Ba*. *caryeae*, *Ba*. *dorsalis*, *Ba*. *invadens, Ba*. *kandiensis*, *Ba*. *occipitalis*, *Ba*. *papayae*, and *Ba*. *philippinensis* form the most serious group of pests of fruits and vegetables. Males are strongly attracted to and compulsively feed on ME, which acts as a) a sex pheromone precursor in *Ba*. *dorsalis* and *Ba*. *papayae*— the latter shown to be neither distinct biological nor genetic species from the former (Naeole and Haymer 2003; Tan 2003; Zimowska and Handler 2005), in which ME is converted mainly to (E)-coniferyl alcohol and 2-allyl-4,5-dimethoxyphenol (Nishida et al. 1988; Tan and Nishida 1996, 1998; Hee and Tan 2004); and b) a booster component to endogenously produced sex pheromone in *Ba*. *carambolae*, where it is biotransformed to only (E)-coniferyl alcohol (Tan and Nishida 1998; Wee et al. 2007). Recently, it was reported that the extremely invasive species in Africa, *Ba*. *invadens*, and in the Philippines, *Ba*. *philippinensis*, convert consumed ME to the same ME metabolites in similar ratio as *Ba*. *dorsalis*, and they belong to the same species clade, while *Ba*. *zonata* biotransformed ME to 2-allyl-4,5-dimethoxyphenol and (Z)-coniferyl alcohol, and *Ba*. *correcta* to (Z)-3,4-dimethoxycinnamyl alcohol and (Z)-coniferyl alcohol (Tan et al. 2011 a,b).

Consumption of ME has been shown to significantly improve male mating competitiveness in *Ba*. *dorsalis* (Shelly and Dewire 1994, 2000; Tan and Nishida 1996, 1998), *Ba*. *carambolae* (Wee et al. 2007), *Ba*. *correcta* (Orankanok et al. 2009), and *Ba*. *zonata* (Quilici et al. 2004; Sookar et al. 2009). Wild fruit fly males have easy access to natural sources of ME (Tan 2009). Therefore, it would be desirable to feed sterile males with ME in order to compete with wild males “on a level playing field”, before mass release so as to enhance mating success in a sterile insect technique (SIT) program (Shelly et al. 2010).

#### *3.3.1.2. Beneficial insect species*. 

The green lacewing, *Ankylopteryx exquisite*, was attracted to ME—baited traps set up in two locations in central Taiwan in large numbers (350–800 adults/trap/two weeks during July) (Pai et al. 2004). Additionally, adults of another lacewing, *Chrysopa basalis*, were captured in plastic traps containing ME (Suda and Cunningham 1970). The reason for their attraction to ME for these predatory insects is still unclear. This is also the case for the weak attraction of honeybees, *Apis mellifera*, to traps baited with ME in high elevation native forest in Hawaii. The number captured varied with seasons, and it was found that more honeybees were captured in March and between June and August (Asquith and Burny 1998). The numbers trapped certainly did not reflect capture due to chance. Therefore, could the worker honeybees be mistakenly guided into ME traps through previously learned odor of ME resembling floral fragrance of golden shower or other flowers (see below)? Perhaps this question may be satisfactorily answered through proper electrophysiological and chemoecological investigations.

### 3.4 Methyl eugenol in flowers—ME as attractant and floral reward

Many plants, besides fending off insect herbivores, may require insects to assist in pollination. Recently, Knudsen et al. (2006) reviewed many aspects of floral scent with respect to variation within and between congeneric species belonging to a genus. They listed 12 common compounds, namely limonene, (E)-ocimene, myrcene, linalool. aand b-pinene, benzaldehyde, methyl 2-hydroxybenzoate, benzyl alcohol, 2-phenylethanol, caryophyllene, and 6-methyl-5-hepten-2-one that are detected in floral scent from over 50% of seed plant families, and also provided a list of 1719 compounds identified from floral fragrances. ME was among the compounds listed and was detected in 21 plant families. Nonetheless, many more plant species produce flowers that possess ME that may be released as a component in floral fragrance. Table 2 shows ∼122 species from 42 plant families, many of which (∼85 species from 22 families) have ME detected exclusively in flowers or floral fragrances. This further substantiates the notion that synthesis of floral ME evolved independently in different plant families and orders. However, 27 species, namely *Cuminum cyminum*, *Daucus carota*, *Pimpinella affinis*, and *Scandix iberica* (Apiaceae), *Achillea conferta*, *Solidago odora,* and *Tagetes lucida*, (Asteraceae), *Borago* officialis (Boraginaceae), *Medicago marina* (Fabaceae), *Agastache foeniculum*, *Ocimum basilicum*, *O*. *gratissimum*, *O*. *sanctum*, *O*. *selloi*, *O*. *suave*, and *Rosemarinus officionalis* (Lamiaceae), *Laurus nobilis* (Lauraceae), *Michelia alba* (Magnoliaceae), *Myrtus communisand* and *Syzygium aromaticum* (Myrtaceae), *Piper betel* (Piperaceae), *Cymbopogon flesuosus* (Poaceae), *Rosa damascena* and *R*. *hybrida* (Rosaceae), *Tamarix boveana* (Tamaricaceae), *Daphne genkwa* (Thymelaceae), and *Lippia alba* and *Lippia schomburgkiiana* (Verbenaceae) also have ME detected in other plant parts (Tables 1 and 2).

Except for several species, neither the role of ME in flowers nor the attraction of fruit flies was mentioned in the published articles. However, if ME is released naturally in an area where *Bactrocera* fruit flies are present, the flowers would have attracted the ME— responsive *Bactrocera* species.

Much of the published work on floral chemical composition with detected ME did not indicate the type of floral visitors or pollinators. While some species of *Dianthus* (Caryophyllaceae) had flowers that bloom at night, these flowers attracted nocturnal insects, such as moths, and bats as visitors/pollinators (Jurgens et al. 2003). Mediterranean flowers of *Dianthus arenarius*, *D*. *monspessulanus*, *D*. *superbus*, and *Silene officinalis* are whitish in color and strongly scented (especially during the night), indicating pollination by night—active flower visitors. Another species, *Silene latifora*, in the same family bears night flowers. The flowers from a European population had no detectable ME, whereas those collected from some plants in a North American population had detectable ME. However, the flowers did not exclude diurnal flower visitors, because unlike some nocturnal *Silene* species, they did not close or wilt during the day following anthesis. Nevertheless, there were clear differences in the floral scent of diurnal butterfly—flowers and moth— or hawkmoth— pollinated nocturnal species. According to Jurgens et al. (2003), the phenylpropanoids such as ME, methyl isoeugenol, elemicin, (Z)-asarone, and (E)-asarone were only found in the nocturnal *Dianthus* species.

Flowers from other families, similar to those of the family Caryophyllaceae, may attract other insects in regions/countries without ME—responsive *Bactrocera* species. Therefore, these flowers are not specifically adapted to fruit fly pollinators even though they possess ME.

#### 3.4.1. ME in flowers with unknown purpose. 

From 16 *Clusia* species (Clusiaceae) under four different taxonomic sections, only two species, *C. parviflora* (section Criuva) and *C. renggerrioides* (section Corylandra) possessed floral ME (Nogueira et al. 2001). The role of ME in the two species is still unknown. This is similar to the often—cited flowers of golden shower or Indian labernum, *Cassia fistula*, that contained ME and attracted the oriental fruit fly, *Ba*. *dorsalis* (Kawano et al. 1968). Recently, the flower essential oil was reported to contain ME at 7.3% of peak areas and trace amount of eugenol; these compounds were not detected in leaf oil (Tzakou et al. 2007). Unfortunately, there is still no report that the attracted fruit flies are either potential pollinators or just visitors.

*Cymbopogon flexuosus* (Poaceae) exists as four varieties based on the major component among approximately 75 constituents in inflorescence essential oils. The varieties of *C*. *flesuosus* (var. *arunachalis*, var. *assamensis*, and var. *sikkimensis*) had citral, citronellol, elemicin, and ME as the major component, respectively. The first two varieties did not possess floral ME. The var. *sikkimensis* had 32–34% floral ME, while var. *assamensis* had 0.2–0.4% of essential oils (Nath et al. 2002). As such, the former variety would be more attractive to ME—responsive *Bactrocera* species than the latter. Nevertheless, this attraction of fruit flies as either pollinators or visitors remains to be determined for the two varieties. This is expected as most floral fragrances contain many chemical components (sometimes well over a hundred), and to ascribe the actual role for each of the ingredients, especially those in trace quantities, is extremely difficult, time consuming, and often unrewarding.

In the family Orchidaceae, many species are known to have trace quantities of ME. Since some of them are known to exist in regions with no insect species that are specifically attracted to ME or flowers in the night (Table 2), it is obvious that the ME—sensitive *Bactrocera* species play no role in pollination. However, flowers of the Malayan type of *Phalaenopsis violacea* possess trace quantities of ME and eugenol (Kaiser 1993), and usually attract one to several fruit flies per flower. The trace amount of floral ME is sufficient to attract fruit flies, since ∼ 1 nanogram (10-^9^g) of ME spotted on a silica gel TLC plate placed in the field can attract native male flies of the ME—sensitive species, such as *Ba*. *dorsalis* (Tan and Nishida 2000). The Bornean type of this orchid species, which is currently placed as a different species, *P*. *bellina*, has none of the phenylpropanoids (Kaiser 1993), although their flowers appear very similar in terms of color pattern and morphology to the untrained eye. As such, the observed attraction of fruit flies to *P*. *bellina* was probably due to the presence of 2,6-dimethoxy-4-(2-propenyl)-phenol. This compound was emitted as a component of floral fragrance at a rate of 12.0 ± 8.5 ng/flower/hour (Hsiao et al. 2006). It is an isomer of 2-allyl-4,5-dimethoxyphenol, which is a relatively strong fruit fly attractant and a component of the oriental fruit fly sex pheromone after ME consumption. Interestingly, *P*. *violacea* has no special adaptation, such as a movable lip as in *Bulbophyllum* orchids (see section 3.4.2.2 B), to aid in the removal of pollinarium (a composite structure of pollinia containing numerous pollens, a tegula/hamulus stipe, and visidium). This is further substantiated by our observations that the ME—sensitive fruit fly males never removed pollinarium from flowers of *P*. *violaceae*, are mere visitors, and thus do not assist in pollination for this orchid species.

It has been proposed that an additional role of floral fragrance may be in defense to deter or repel insect herbivores/florivores, as many of the floral volatile compounds are also released from leaves in response to herbivore damage (Kessler and Baldwin 2001). This is further substantiated by ME, which is used by plants as a chemical defense as previously discussed in section 3.2. Therefore, floral ME, which appears not to have any specific function in pollination, may be playing a ‘silent’ role in deterring and/or repelling possible insect florivores.

#### 3.4.2. In pollination. 

Floral fragrance is presumably for the sole purpose of guiding potential pollinators to perform pollination that results in fertilization of flowers. The presence of ME in floral fragrances, even in trace quantities, may be responsible for attracting potential *Bactrocera* pollinators in the tropical/subtropical regions where the ME—responsive species of fruit flies are endemic.

##### 3.4.2.1. For non—orchid flowers

**Figure 3.**
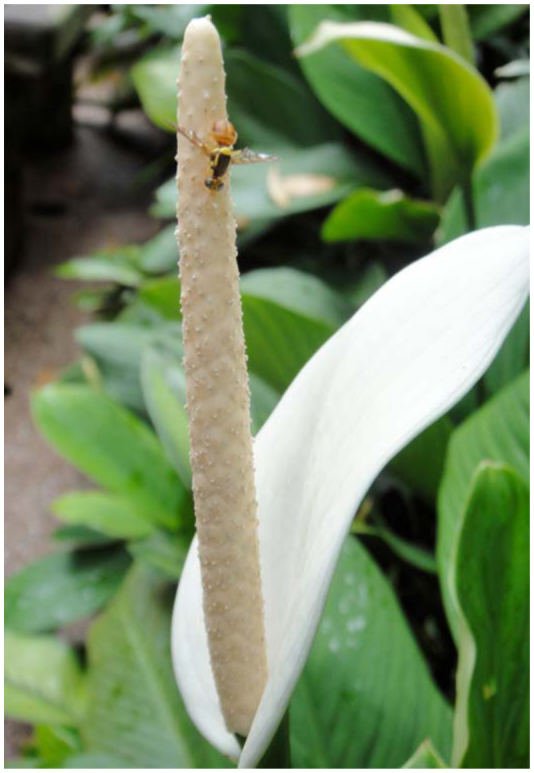
A male *Bactrocera* umbrosa feeding on *Spathiphyllum cannaefolium spadix.* High quality figures are available online.

The fruit fly lily *Spathiphyllum cannaefolium* (Araceae) floral spadix has a high content of ME (Lewis et al. 1988), which attracts many ME—sensitive *Bactrocera* male flies to visit and pollinate the flower by transferring white powdery pollens as the flies feed on the spadix. Plants grown in Penang (Malaysia) often attract one or two fruit fly males (Figure 3) as well as stingless bees (*Trigona* species) for pollination (unpublished observation).

Another Araceae species, *Colocasia esculenta*, which contained ME and eugenol (relative quantities not provided), attracted many male *Ba*. *dorsalis* fruit flies (> 40) to the spadix and bract (Sinchaisri and Areekul 1985). In this species, only the fruit flies feeding on the spadix will pick up powdery pollens and transfer them to the stigmas on the radix.

Flowers of the cannon ball tree *Couroupita guinanensis* (Lecythidaceae) contained 3% eugenol with a trace quantity of ME in floral essential oil (Knudsen and Mori 1996). Flowers in tropical South America have been observed to attract many male *Ba*. *carambolae* fruit flies in Suriname (photograph shown by van Sauers-Muller, personal communication, 2010). However, the flowers obtained from trees grown in the Botanical Garden in Penang have eugenol and no detectable ME, and they attract many stingless bees (*Trigona* species) with an occasional *Ba*. *dorsalis* as a visitor (unpublished observation).

Paraguay jasmine, *Brunfelsia australis* (Solanaceae), commonly known as “Yesterday—Today—and—Tomorrow”, has floral fragrances comprised of monoterpenoids (81% of the identified volatile compounds), with ME in trace quantity in young flowers and 0.1% content of mature flowers. But in the scentless mature flowers of a closely related species, *Brunfelsia pauciflora* (Fabaceae), two sesquiterpenes (γmuurolene and α-copaene) were present with no detectable ME (Bertrand et al. 2006). Similarly, the only species in the Onagraceae family that emits a floral scent containing substantial ME is *Clarkia breweri* (Table 2); its closely related *Clarkia concinna* is virtually scentless with no detectable ME (Raguso and Pichersky 1995).

##### 3.4.2.2. For orchid flowers

Orchids have evolved highly diverse and fascinating mechanisms to attract and entice animals, especially insects, to assist in cross— pollination. In this section, discussion will be confined to orchid flowers that possess or secrete ME that attracts insects to be pollen vectors.

###### 3.4.2.2a. Orchids excluding 

*Bulbophyttum*. Orchid flowers of *Satyrium microrrhynchum* produce nectar and are visited by several species of flower—visiting insects such as beetles, wasps, and flies, but not various honeybees and solitary bees that are commonly present at the study sites. Two insect species, cetoniid beetles, *Atrichelaphinus tigrina* (both sexes) and a pompilid wasp, *Hemipepsis hilaris* (males), have been shown to be pollinators while the other insect visitors do not carry any pollinarium (Johnson et al. 2007). Linalool is the major chemical component in the orchid fragrance and has been shown to attract the pollinators. Although seven phenylpropanoids with ME (at 1.83–4.51%) as the highest component were detected in the flowers from one of three populations studied in South Africa, there was no difference in the type of insect visitors/pollinators observed, as ME also stimulated an electrophysiological response in antennae of the cetoniid beetle (Johnson et al. 2007).

The inflorescence of an orchid species, *Gymnadenia conopea*, emits both eugenol and ME at different relative quantities during the day and night (Table 2). It attracts six lepidopteran taxa: three species each of butterflies and moths. Among the lepidopteran visitors caught, two species each of butterflies and moths bore pollinia. This indicates that pollination occurs during the day as well as at night (Huber et al. 2005). Similarly, a closely related species, *Gymnadenia odoratissima*, has 10 lepidopteran taxa, six moth, and four butterfly species as floral visitors, and all the species have been observed to be pollinators confirmed via their bearing of pollinia. There is no overlap of pollinator species between the two orchid species, and eugenol and benzyl acetate, which are among several of the 44–45 volatiles, are physiologically active components in the floral scent of the two species (Huber et al. 2005). In these orchid species, ME is not physiologically active against the lepidopteran species attracted to the orchid flowers and may instead be playing a role in deterring florivores. This certainly warrants further investigation.

###### 3.4.2.2b. Bactrocerophilous *Bulbophyttum *orchids. 

There are nearly 2000 recognized species of *Bulbophyllum* (Orchidaceae) worldwide. Some species (∼30) are known to have adapted to, and are entirely dependent on, *Bactrocera* (Tephritidae: Diptera) fruit flies for pollination without offering the usual nectar as floral reward. These bactrocerophilous *Bulbophyllum* species might have coevolved with the tephritid fruit flies. They basically make use of either RK, detected in *Bu*. *apertum* (syn. *Bu*. *ecornutum*) (Tan and Nishida 2005), zingerone in *Bu*. *patens* and *Bu*. *baileyi* (Tan and Nishida 2000, 2007), or ME (examples given below) as a floral attractant and reward for male *Bactrocera* fruit flies (Tan 2009). It is interesting to note that zingerone is the only known compound to attract both RK— and ME—responsive *Bactrocera* species, although it is a relatively weak attractant due to its resemblance to both RK and ME chemical structures (Tan and Nishida 2000).

**Figure 4.**
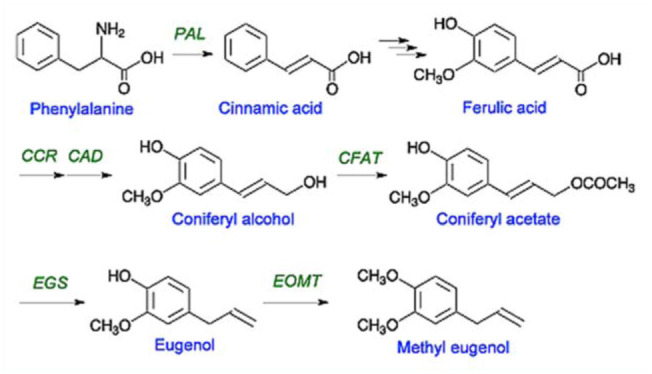
A possible biosynthetic pathway of methyl eugenol in an orchid flower of a bactrocerophilous *Bulbophyllum* species. **PAL**, phenylalanine ammonia lyase; **CCR**, cinnamoyl-CoA reductase; **CAD**, cinnamyl alcohol dehydrogenase; **CFAT**, coniferyl alcohol acyltransferase; **EGS**, eugenol synthase; **EOMT**, eugenol O-methyltransferase. High quality figures are available online.

The possible pathway for the biosynthesis of ME found in *Bulbophyllum* is shown in Figure 4. Starting from phenylalanine, it undergoes a series of intermediary steps involving cinnamic acid, ferulic acid, coniferyl alcohol, coniferyl acetate, and eugenol (Figure 4) (Kapteyn et al. 2007; Ferrer et al. 2008). The eugenol is ultimately biotransformed to ME by the addition of a methyl group to the ‘para— hydroxy’ group of eugenol catalyzed by an O-methyltransferase (Lewinsohn et al. 2000; Pichersky and Gang 2000).

**Figure 5.**
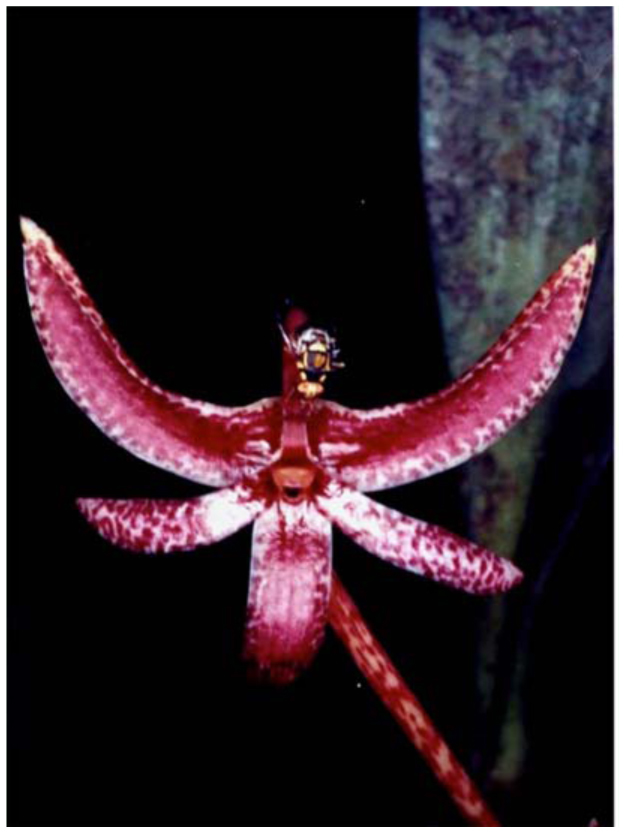
A male *Bactrocera dorsalis* bearing pollinia on see—saw lip of *Bulbophyllum* patens. High quality figures are available online.

Here only *Bulbophyllum* flowers that possess and release ME as a component of floral fragrance will be discussed to show that the flowers of some species have coevolved, via special floral architectural modifications to enhance fly pollination, with *Bactrocera* male flies. A nonresupinate flower (with lip/labellum above the floral column) of the ginger orchid, *Bu*. *patens*, possesses a major component of a fruit fly attractant, zingerone, which is weakly attractive to *Bactrocera* males from both ME—responsive species, such as *Ba*, *carambolae*, *Ba*. *dorsalis* and *Ba*. *umbrosa*, as well as RK—responsive species, namely *Ba*. *caudata*, *Ba*. *Cucurbitae*, and *Ba*. *tau*, with trace amounts of ME (Tan and Nishida 2000). It has a see—saw lip that is positioned in a plane above the floral column. When an attracted male *Ba*. *dorsalis* alights on and continues feeding along the lip, an imbalance will occur, and the fly will suddenly be tipped into the column cavity head first. The fly immediately retreats by moving backwards along the lip still in a closed position, and during this movement it removes the pollinia to initiate pollination. This process is repeated when a fly bearing pollinia lands on another flower (Figure 5) to initiate fertilization by depositing the pollinia onto the stigma.

**Figure 6.**
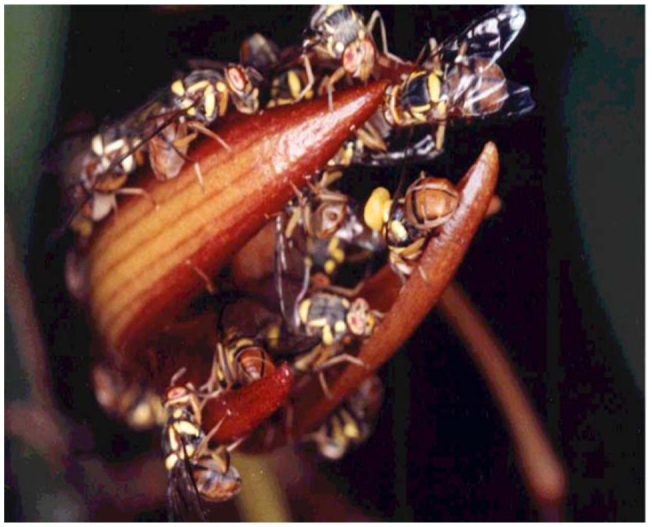
Male fruit flies, *Bactrocera dorsalis*, congregating and licking on a fully bloomed *Bulbophyllum cheiri* flower. High quality figures are available online.

**Figure 7.**
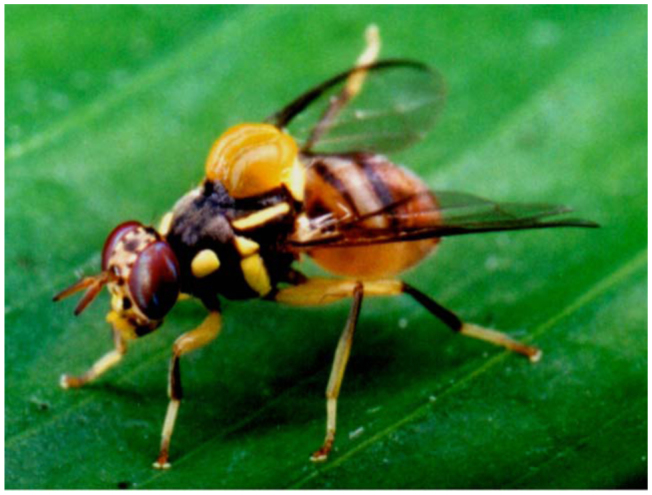
Male *Bactrocera dorsalis* bearing pollinia of *Bulbophyllum cheiri.* High quality figures are available online.

The fruit fly orchid, *Bulbophyllum cheiri*, with non—resupinate and a solitary flower, does not have its sepals and petals fully spread out but just slightly parted when fully in bloom (Figure 6). It releases ME as its sole major volatile component in its floral fragrance, which attracts only male fruit flies (Tan et al. 2002). The concentration of ME in the various floral parts varies from 107, 95, 91, 44, and 41 ppm for lateral sepals, lip, petals, median sepal, and column, respectively (Tan et al. 2002). Further surveys identified seven more related analogs, including eugenol, (Z)-methyl isoeugenol, (E)-methyl isoeugenol, (E)-coniferyl alcohol (CF), 2-allyl-4,5-dimethoxyphenol (DMP), 5-allyl-1,2,4-trimethoxybenzene (euasarone), and (E)-3,4-dimethoxycinnamyl acetate (Nishida et al. 2004). It is interesting that the two major sex pheromonal components of *Ba*. *dorsalis*, CF and DMP, are also found in the orchid flowers. Many male flies of *Ba*. *dorsalis* with one or two *Ba*. *umbrosa* visit a newly bloomed flower in the morning. Usually, the first fly visitor removes the pollinia from the flower (Figures 6 and 7). Here the movable floral see—saw lip plays an important role in suddenly tipping a probing fly into the floral column cavity when an imbalance occurs due to the shifting of the fly's weight. This way the fly, during its retreat, either removes or deposits pollinia on the floral stigma. Headspace analysis of the flower indicates a high ME peak in the morning, a much smaller one between 12:00 and 14:00, and no ME detected after 14:00 (Tan et al. 2002). In spite of this, one or two male *Ba*. *dorsalis* flies can still be seen on a *Bu*. *cheiri* flower up until approximately 18:30 (personal observations).

**Figure 8.**
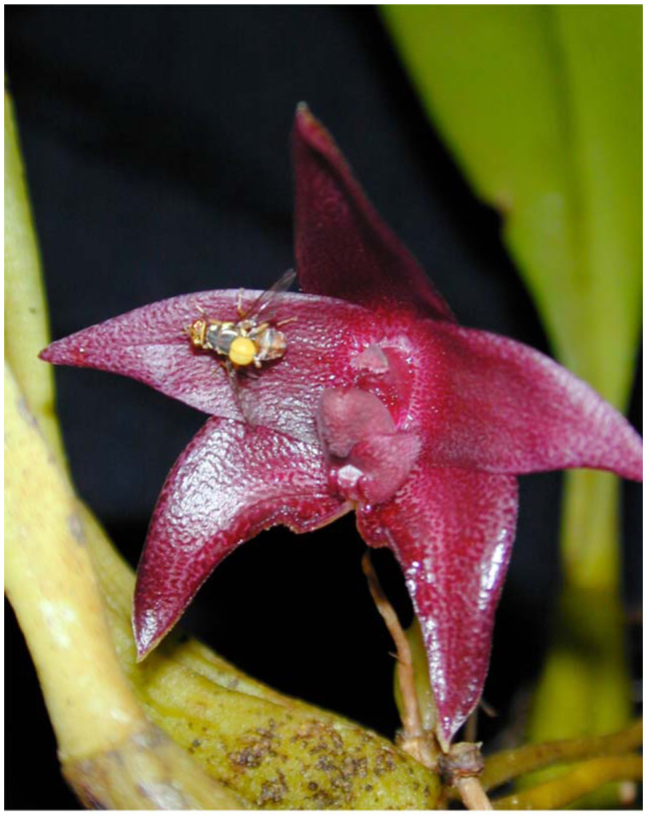
Flower of *Bulbophyllum vinaceum* with its spring— loaded lip in a closed position and a pollinarium—bearing fruit fly, *Bactrocera dorsalis.* High quality figures are available online.

The wine red orchid, *Bu*. *vinaceum*, bears resupinate (lip/labellum below the floral column) and a solitary flower, which has a spring—loaded lip kept in a closed position to protect its sexual organs, especially the pollinarium with a stiff hamulus (derived from the entire distal portion of the rostellum that is prolonged into a stalk). The major floral volatile components identified are ME, CF, DMP, and (E)-3,4-dimethoxycinnamyl acetate, whereas the minor components are eugenol, euasarone, (E)-3,4-dimethoxy cinnamyl alcohol, and (Z)-coniferyl alcohol. The bouquet of floral phenylpropanoids attracts ME—sensitive species, particularly *Ba*. *dorsalis* with one or two *Ba*. *unimacula* in the highlands of Sabah (Tan et al. 2006). An attracted male fly normally lands on one of the petals before climbing onto and forcing the “spring loaded” floral lip that has the highest concentration of the phenylpropanoids, into the open position. This action reveals the floral sexual organs. The architecture of the lip and location of attractants compel the fly to align itself precisely along the lip's longitudinal axis. As the fly probes and feeds, it passes the point of imbalance, causing the lip to spring back to its normal closed position. This catapults the fly head first into the column cavity, and its dorsum strikes the protruding sticky base of the hamulus and adheres to it. The momentum of the fly and the structural morphology of the long stiff hamulus act in tandem to pry out the pollinia from its anther cover. Pollinarium removal (Figure 8) is a precise and very quick process assisted by the specially modified spring lip, which plays an essential and important role in pollination. In this orchid species, ME is the main component in the floral fragrance and plays a pivotal role in the true mutualism between the flower and fruit fly pollinator, in which both receive reproductive benefits. Interestingly, both CF and DMP detected in the flowers are also sex pheromonal components of male *Ba*. *dorsalis* after consuming ME. Although CF and DMP attract and arrest females during courtship at dusk, and thus would serve as specific female attractants, the flower has never been observed to attract female fruit flies, not even during dusk when they are most sensitive to these chemicals (Tan et al. 2006). This evidence, and that of *Bu*. *cheiri*, may substantiate and indicate the outcome or culmination of a co—evolutionary process between the orchid species and *Bactrocera* pollinators.

**Figure 9.**
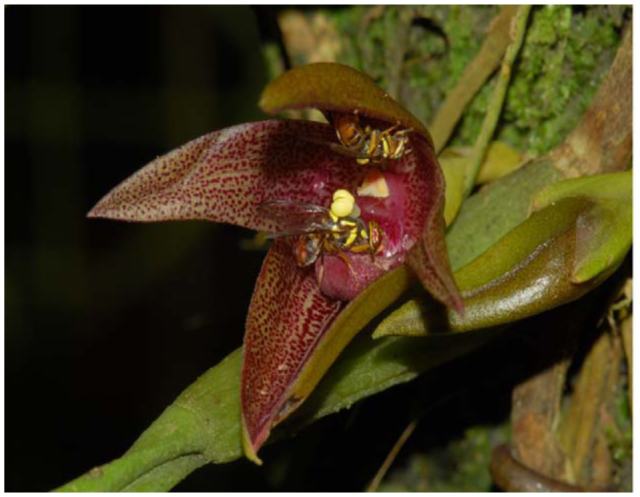
A male *Bactrocera dorsalis* bearing a pollinarium just removed from the *Bulbophyllum elevatopuntatum* flower (P.T. Ong). High quality figures are available online.

The ‘raised dot *Bulbophyllum’*, *Bu*. *elevatopunctatum,* has relatively high content of ME 78.5 ±+ 21.6 mg (mean + standard deviation; n= 10) per flower as a major floral volatile (unpublished data). The solitary and resupinate flower does not have a spring— loaded lip like that present in *Bu*. *vinaceum*, but a simple hinged one kept at an acute angle with respect to the floral column by the fused lateral sepals. When an attracted male fruit fly moves on to the lip that is prevented from moving away from the column to a fully opened position, it will very quickly be jerked into the floral column cavity, thereby hitting the hamulus and dislodging the pollinia from the anther and its cover. Upon its retreat, the fly removes the pollinarium to initiate pollination (Figure 9).

In the aforementioned *Bulbophyllum— Bactrocera* association, each *Bulbophyllum* species has specifically adapted and evolved precise lip mechanism to entice fruit flies and enhance pollination through the offer of ME as an attractant as well as a floral reward. Furthermore, both organisms gain direct reproductive benefits, exhibiting a true mutualism; the orchid flower gets pollinated without having to offer nectar as reward, and the fruit fly boosts its pheromone and defense system as well as its sexual competitiveness by feeding on the ME produced by the flower as floral reward to its potential pollinator.

## 4. Methyl eugenol and human health

When present in human blood serum after a meal, ME is rapidly eliminated and excreted (Schecter et al. 2004). ME has ill effects on human health as a known carcinogen and mutagen, probably because of its conversion to a hydroxy analog at the allylic position (De Vincenzi et al. 2000). Further, safrole, estragole, and ME found in herbs and spices are weak animal carcinogens as demonstrated by the formation of DNA adducts in cultured human cells (Zhou et al. 2007).

Recent research by Choi et al. (2010) indicated that ME may have positive effects on human health as well. Based on their studies, ME may reduce cerebral ischemic injury through suppression of oxidative injury and inflammation (Choi et al. 2010). The chemical also decreased activation of an enzyme, caspase-3, and the death of cultured cerebral cortical neurons through oxygen— glucose deprivation for one hour. Additionally, it was shown that ME elevated the activities of superoxide dismutase and catalase, thereby markedly reducing superoxide generation in the ischemic brain and decreasing intracellular oxidative stress. Furthermore, ME also reduced the production of pro—inflammatory cytokines in the ischemic brain (Choi et al. 2010).

Studies on rodents showed that minimal ME within a dose range of 1–10 mg/kg body weight, which is about 100–1000 times the anticipated human exposure to ME as a result of spiced and/or flavored food consumption, did not pose a significant cancer risk (Smith et al. 2002). Further, toxicological studies in animals demonstrated that orally administered relatively high—bolus doses of ME resulted in hepatic neoplasms. Nevertheless, the detected level of ME in biomonitoring studies indicated that human exposure was several orders of magnitude lower than the lowest dose utilized in the bioassay (Robison and Barr 2006). Arguably, a single high dose may cause any number of ill or side effects in animals.

## Conclusions

In this review, the occurrence of ME in over 450 species of plants belonging to 80 families under 48 orders compiled from numerous published papers is listed. The distribution of ME in various plant organs within a species is definitely uneven and varies greatly according to growth stage as well as plant variety/chemotype. Similarly, even in flowers, the distribution and release of ME by various floral parts can vary considerably depending on the physiological stage and time of day.

The various roles of ME in nature especially related to the chemical defense of plants, such as antifungal, antibacterial, antinematodal, or toxicant roles against pathogens and insect herbivores, as well as its functions as an insect antifeedant/repellant and in pollination are reviewed. In particular, ME has been shown to act as floral synomone in the coevolution of orchid species in the genus *Bulbophyllum* with fruit flies. More research should be conducted to fully understand the biochemical, physiological, and/or chemoecological basis for these bitrophic interactions between plants and insects mediated by ME.

Table 1. Plant family (order) and species containing methyl eugenol (ME)*.

**Table:**
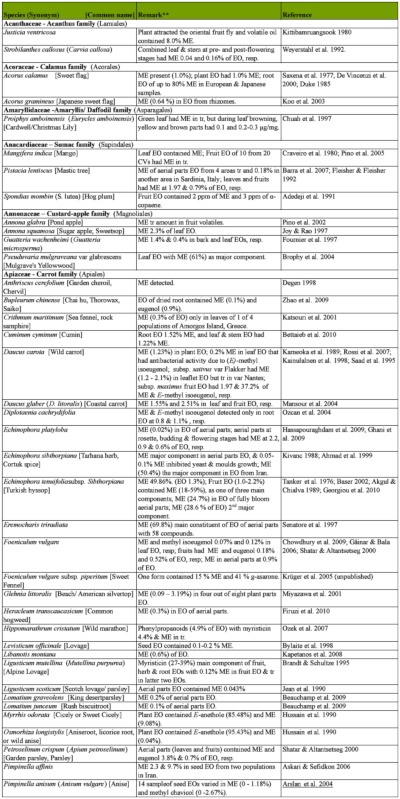

**Continued:**
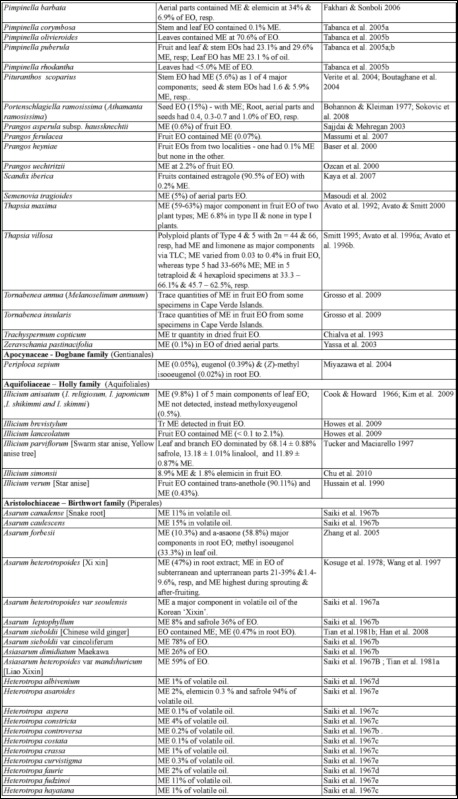

**Continued:**
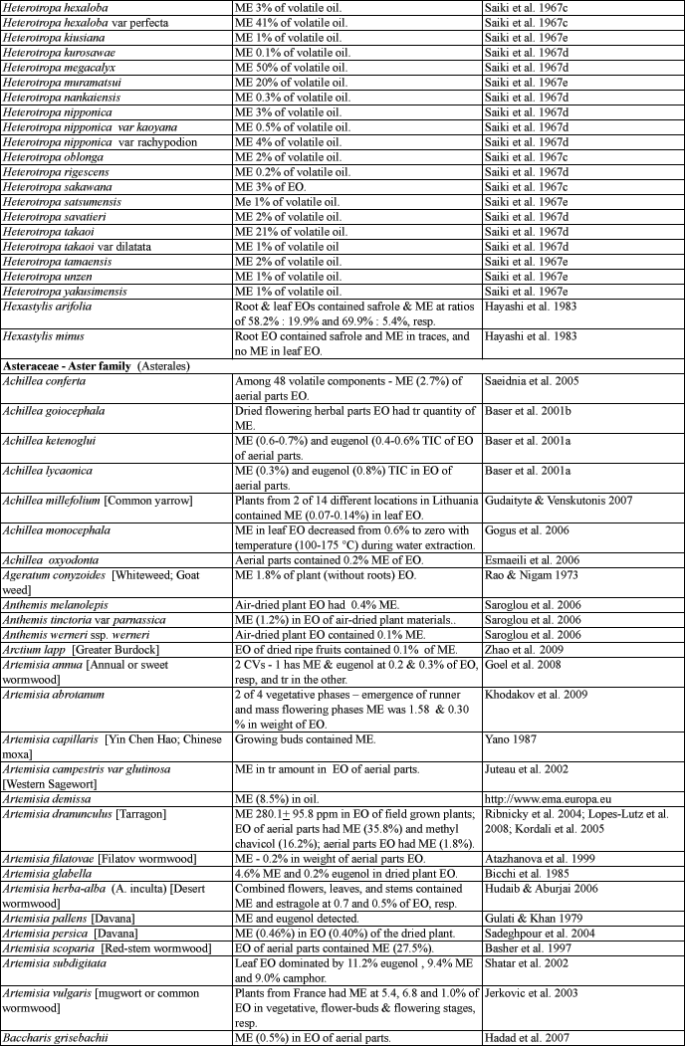

**Continued:**
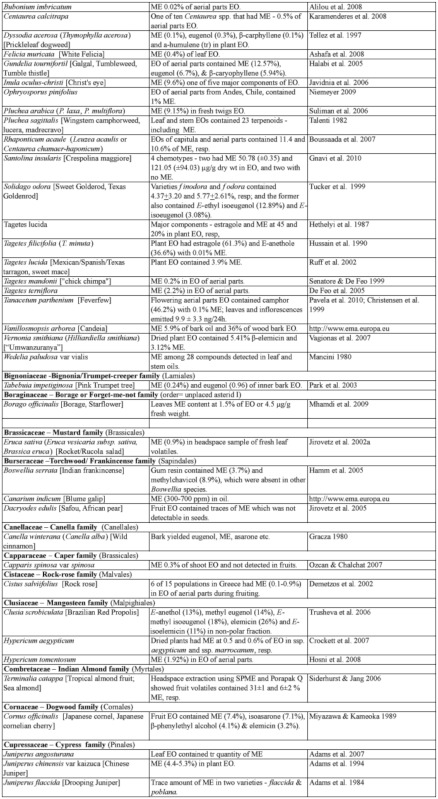

**Continued:**
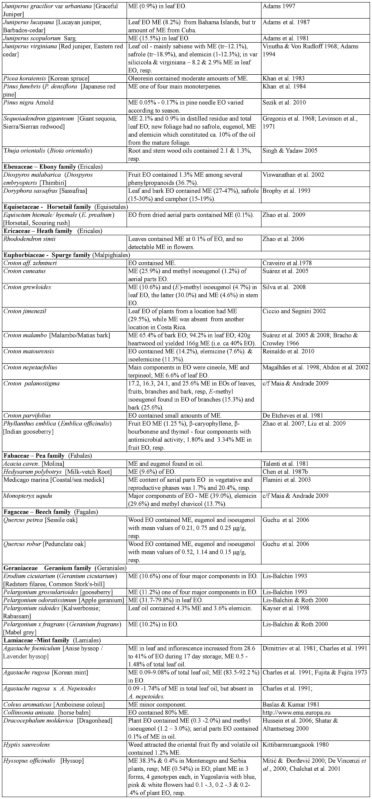

**Continued:**
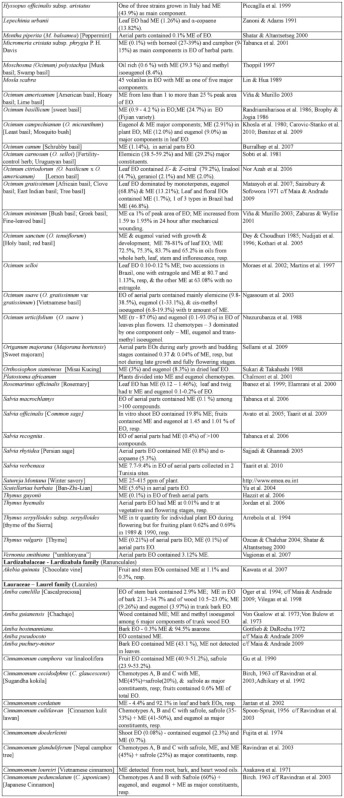

**Continued:**
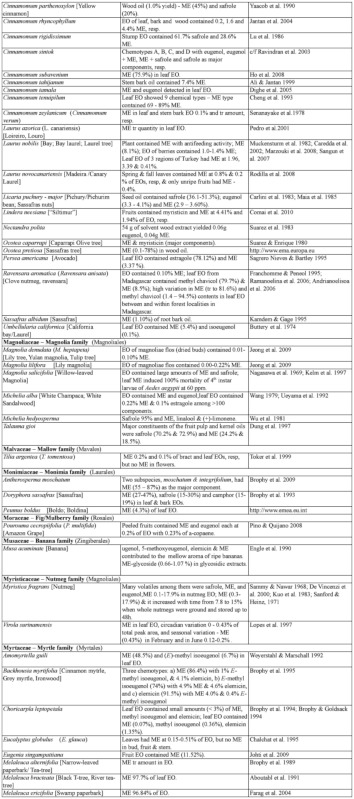

**Continued:**
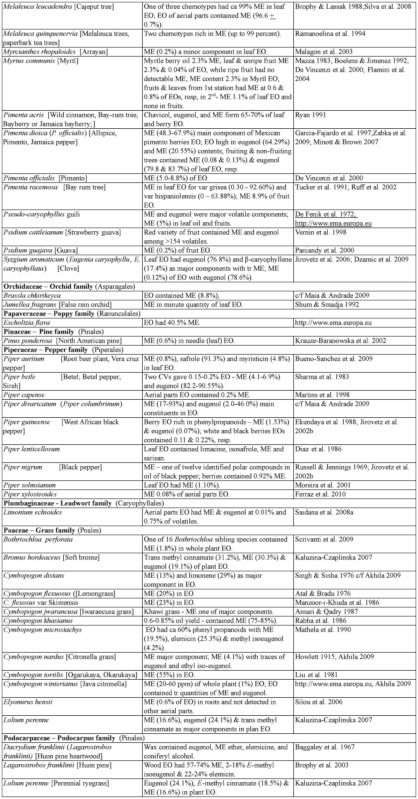

**Continued:**
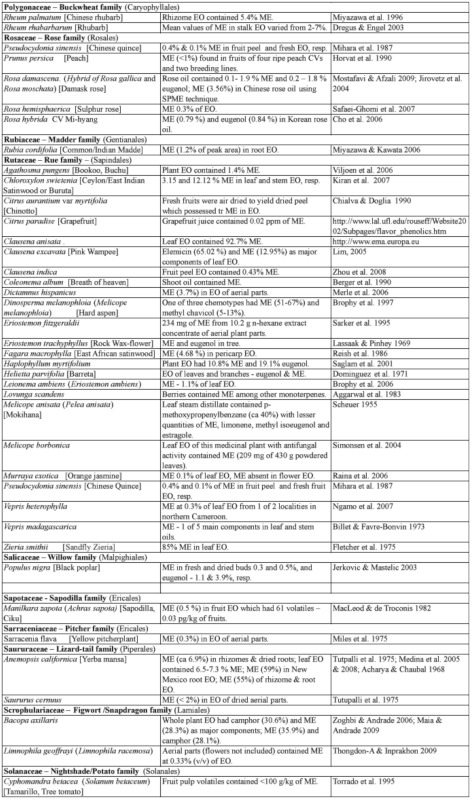

**Continued:**
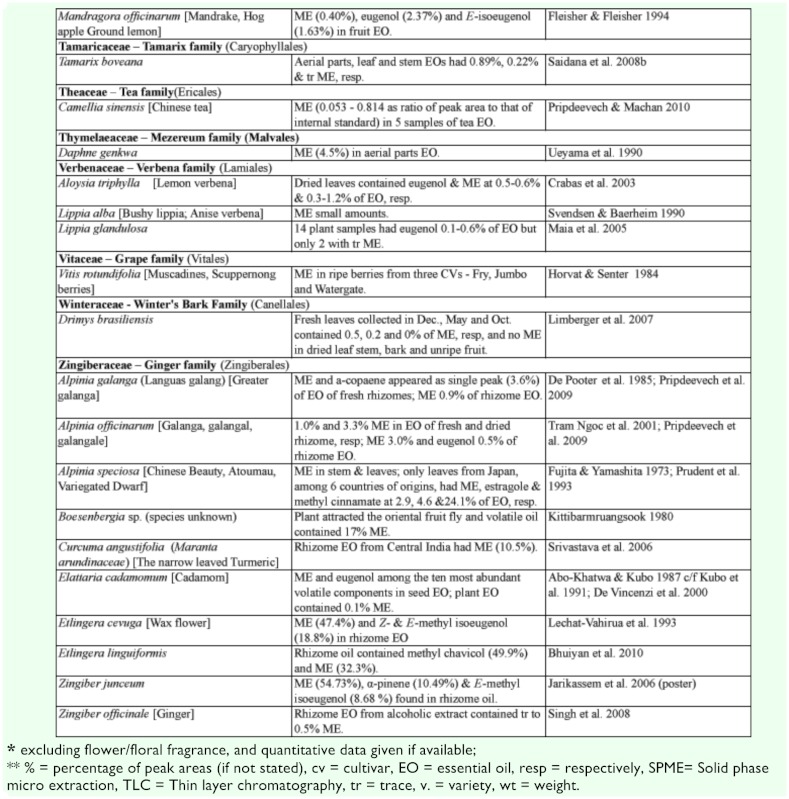

Table 2. Plant family (order) and species containing methyl eugenol [ME] in flowers*.

**Table:**
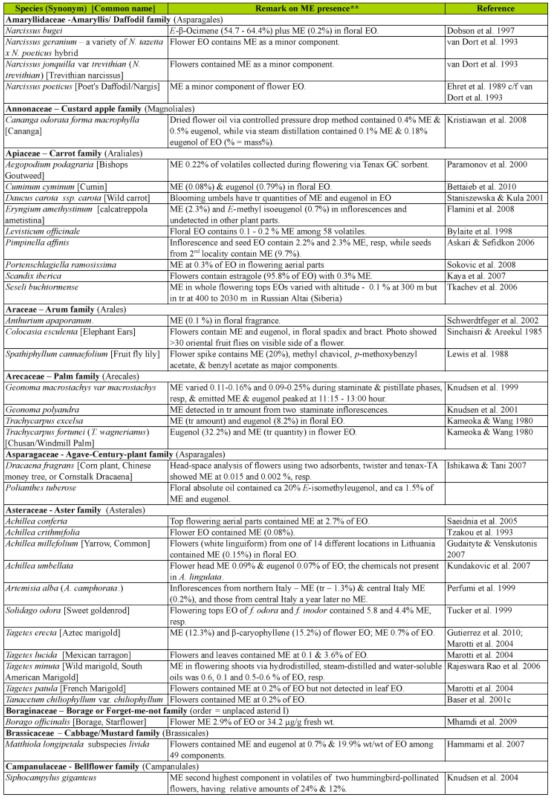

**Continued:**
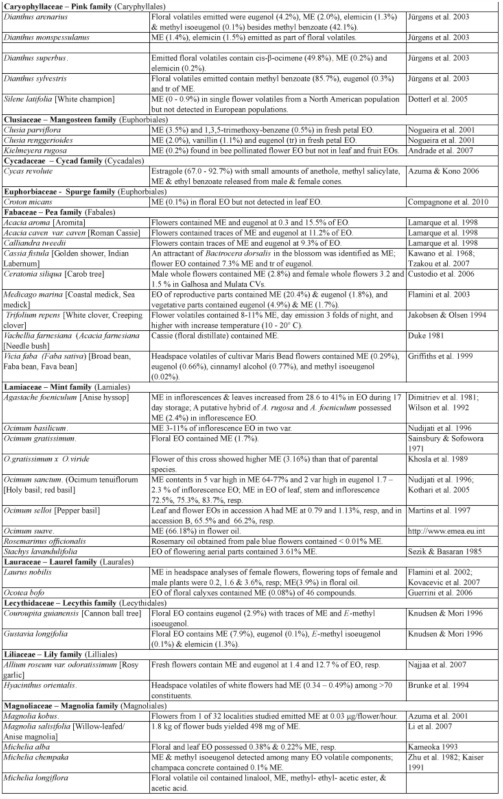

**Continued:**
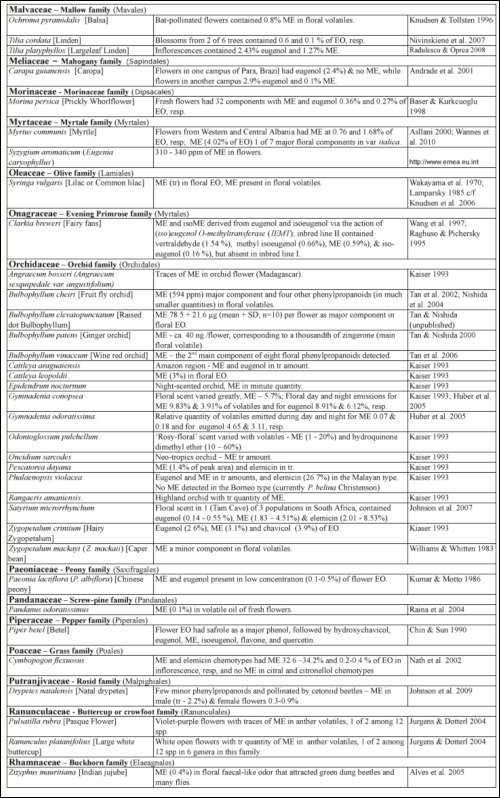

**Continued:**
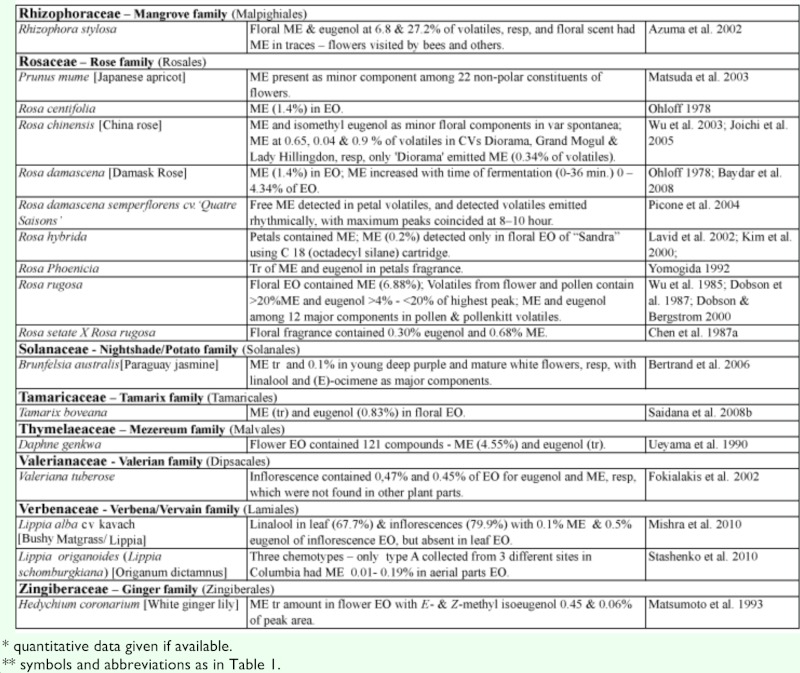

## **Abbreviations**

ME,: methyl eugenol;
RK: raspberry ketone
